# The Fluoroless Future in Electrophysiology: A State-of-the-Art Review

**DOI:** 10.3390/diagnostics14020182

**Published:** 2024-01-14

**Authors:** Alberto Preda, Eleonora Bonvicini, Elena Coradello, Alessio Testoni, Lorenzo Gigli, Matteo Baroni, Marco Carbonaro, Sara Vargiu, Marisa Varrenti, Giulia Colombo, Marco Paolucci, Patrizio Mazzone, Fabrizio Guarracini

**Affiliations:** 1 Electrophysiology Unit, Cardio-Thoraco-Vascular Department, ASST Grande Ospedale Metropolitano Niguarda, 20162 Milan, Italymarco.carbonaro@ospedaleniguarda.it (M.C.); patrizio.mazzone@ospedaleniguarda.it (P.M.); 2 Division of Cardiology, Department of Medicine, Verona University Hospital, 37129 Verona, Italy

**Keywords:** 3D electroanatomic mapping system, ALARA, catheter ablation, fluoroless, intracardiac echocardiography, radiation exposure

## Abstract

Fluoroscopy has always been the cornerstone imaging method of interventional cardiology procedures. However, radiation exposure is linked to an increased risk of malignancies and multiorgan diseases. The medical team is even more exposed to X-rays, and a higher incidence of malignancies was reported in this professional group. In the last years, X-ray exposure has increased rapidly, involving, above all, the medical team and young patients and forcing alternative fluoroless imaging methods. In cardiac electrophysiology (EP) and pacing, the advent of 3D electroanatomic mapping systems with dedicated catheters has allowed real-time, high-density reconstruction of both heart anatomy and electrical activity, significantly reducing the use of fluoroscopy. In addition, the diffusion of intracardiac echocardiography has provided high anatomical resolution of moving cardiac structures, providing intraprocedural guidance for more complex catheter ablation procedures. These methods have largely demonstrated safety and effectiveness, allowing for a dramatic reduction in X-ray delivery in most arrhythmias’ ablations. However, some technical concerns, as well as higher costs, currently do not allow their spread out in EP labs and limit their use to only procedures that are considered highly complex and time-consuming and in young patients. In this review, we aim to update the current employment of fluoroless imaging in different EP procedures, focusing on its strengths and weaknesses.

## 1. Introduction

Fluoroscopy is an imaging technique that uses X-rays to obtain real-time moving images of the human body organs and, so far, has been the gold standard imaging guidance for most interventional cardiology procedures. Radiological exposure is a hot topic nowadays, as cardiology is responsible for about 40% of the entire exposure from all medical sources [1,2]. In electrophysiology (EP), fluoroscopy leads the majority of the procedures, being the only imaging guidance available for cardiac pacing procedures and still a cornerstone for most catheter ablations (CAs) performed into the right chambers. However, the utmost concern of long-term ionizing radiation exposure of both patients and personnel related to the inherent risk of neoplasms pushed scientific research toward alternative imaging methods [3]. Indeed, the U.S. Nuclear Regulatory Commission recommends making every effort to keep exposure to ionizing radiation as low as reasonably achievable (ALARA) [4]. Therefore, in the last twenty years, several non-fluoroscopic imaging technologies have been developed and have slowly taken place in EP labs, dramatically reducing radiation exposure for both the patient and the medical team [5,6,7]. With the advent of fluoroless imaging tools, such as three-dimensional (3D) electroanatomic mapping (EAM) systems and intracardiac echocardiography (ICE), most CA procedures can be performed without X-rays [8]. EAM systems provide consistent advantages compared to fluoroscopy because they allow the building of a real-time, intracavitary 3D map of the heart chambers and the heart’s electrical activity [9]. ICE, by placing an ultrasound probe or transducer directly into the heart chambers, also provides real-time, high-resolution anatomical images, allowing for precise guidance during CA procedures involving moving structures [10]. In several studies, minimal and zero fluoroscopy approaches were demonstrated to be associated with shorter operating time and ionizing radiation exposure without compromising the safety and efficacy of treatments [11]. Their main employment involves CAs of supraventricular tachycardia (SVT) such as atrioventricular nodal reentrant tachycardia (AVNRT), atrioventricular reentrant tachycardia (AVRT), atrial fibrillation (AF), atrial flutter (AFL), and premature ventricular contractions (PVCs), although all EP procedures could potentially be fluoroless. However, some technical factors, the need for experienced operators, and costs limit the spread of their use. In this review, we aim to update the current state of non-fluoroscopic EP procedures, focusing on their strengths and weaknesses.

## 2. Radiation-Related Risks

Interventional cardiologists in EP are one of the occupational categories that are more exposed to radiation [12,13,14]. X-rays are known to be harmful and carcinogenic [15], and the most radiation-sensitive solid organs are the lungs, breasts, colon, bladder, and thyroid, while leukemia is the early diagnosed cancer after radiation exposure. Furthermore, a number of X-ray-related diseases different from cancer, such as dermatitis, cataracts, and cognitive impairment, have also been frequently described [12]. Transient or irreversible infertility and congenital malformations have been demonstrated after X-ray exposure [15]. Radiation exposure carries adverse effects on the human body that are classified as deterministic and stochastic. Deterministic effects are dose-related and defined as a safety threshold over which the severity of harm increases. These effects are directly related to ionizing radiation exposure and its negative effect on cellular repair mechanisms and functions. Dermatitis, cataracts, bone necrosis, and myocardial damage are typical consequences of deterministic effects. On the contrary, the stochastic effect is related to the probability of developing a disease with any amount of exposure in a non-linear way. Accordingly, even a single exposure to ionizing radiation can be associated with carcinogenesis. In this case, DNA damage leading to the activation of oncogenes and oncogenesis pathways underlies the final effect, resulting in cancer. The radiation-induced effects can also be acute or cumulative, and the degree of the injury and the timing depends on the amount of X-ray exposure. Cumulative effects may occur many years after exposure. Of note, cumulative effects are reported worse in obese patients who need increased doses of radiation than normal weight patients [13]. Since a threshold for safe exposure to radiation cannot be defined and no radiation is the most acceptable threshold that can be considered, the American College of Cardiology has stated the importance of reducing ionizing radiation exposure, defining the “As Low As Reasonable Achievable” (ALARA) concept [14,16]. According to ALARA, every procedure involving ionizing radiation needs to be performed without forgetting about the exposure risks. To understand the amount of radiation exposure during a common CA, it must be considered that a radiofrequency (RF) ablation requires a radiation dose from 1 to 25 mSv, while a chest X-ray requires only 0.02 mSv [17]. A 0.1–0.5% increase in fatal malignancy after AF ablation was reported in a study [18]. Moreover, the burden of radiation exposure has significantly increased in recent years due to the increase in the average age of patients and the increase in diagnostic and interventional procedures. On the other part, more and more younger patients are undergoing procedures using X-rays. Finally, the pediatric population must be carefully considered due to increased susceptibility to radiation damage due to the more active cell turnover and long life expectancy [19,20,21]. In particular, children affected by congenital heart diseases are at significant risk of developing stochastic and cumulative effects during their lives because of the constant need for numerous examinations and procedures [22]. In this state, actions to reduce the amount of X-ray exposure are deemed necessary. Healthcare operators have adopted several precautions to protect themselves from X-rays by wearing lead aprons, lead glasses, thyroid shields, and wearable dosimeters and using ceiling- and table-mounted shields. However, radiation exposure never reaches zero [23], and the main source of exposure for the medical team is the scattering radiation arising from the patient [22]. Of particular interest, some studies reported a higher incidence of brain tumors involving the left hemisphere among interventional cardiologists [24,25]. Another non-negligible X-ray-exposure-related consequence is orthopedic diseases, mainly involving the spine and joints, caused by the everyday wearing of lead aprons [22,26]. Chronic neck and back pain, as well as cervical disc herniations, are frequently encountered among operators and are a cause of absences from work and anticipated retirements [27]. Finally, risk exposure is an absolute contraindication for pregnant women, who, therefore, must be removed from their occupation for numerous months. 

## 3. Non-Fluoroscopic Technologies

**Figure 1:**
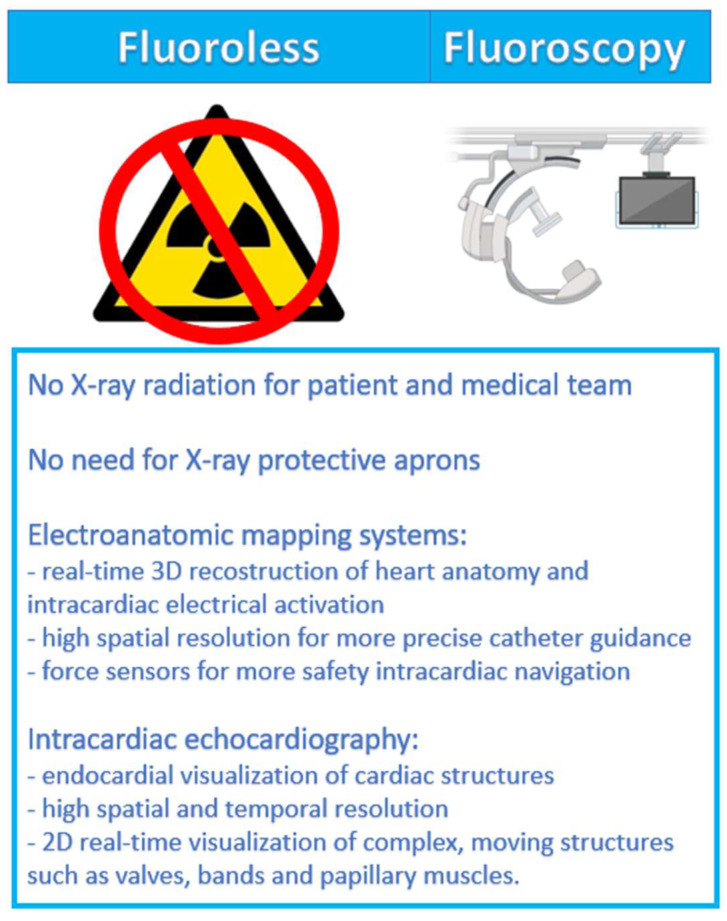
Flouroless procedures.

Radiation exposure reduction in EP procedures started with the introduction of new technologies that can be applied with minimal or zero use of fluoroscopy. Electrical and magnetic fields applied by 3D EAMs and ultrasound technologies used for ICE underwent significant improvements since their first release, leading to new perspectives in the EP lab. Figure 1 compares the use of fluoroscopy with fluoroless technologies.

### 3.1. Three-Dimensional Electroanatomic Mapping Systems

Three-dimensional EAM systems are among the most valuable projects produced by multidisciplinary teamwork that included physicians and engineers and were introduced in the 1990s. They provide a 3D reconstruction of heart structures supplying both anatomical and electrophysiological characteristics of cardiac chambers, such as the activation time, the voltage amplitude, and the presence of late potentials. The spread of EAM systems has given a turning point to the understanding of the characteristics of arrhythmias, in particular, more complex ones such as AF and VT, providing new insights and improving the effectiveness of their treatment. At the same time, different EAMs based on tissue electrical characteristics or potential propagation are generated and integrated [28]. The greater spatial resolution provided by mapping systems associated with the use of contact sensible catheters has improved the safety of procedures, reducing the rate of severe complications according to the complexity of the procedure [29,30]. Different systems based on different mapping technologies have been developed. The CARTO^®^ system (Biosense Webster Inc., Johnson & Johnson (J&J), New Brunswick, NJ, USA) is based on three generators positioned under the table, each of which emits a low-level magnetic field; the location of the mapping catheter is identified by calculating the distance from the three different magnetic fields, and an external pad is applied to the back of the patient to monitor any movement. Specific catheters equipped with electromagnetic sensors are required. Six skin patches are also applied on the patient that allow for the measurement of the level of impedance at the tip of the catheter, which provides useful information both during the mapping and ablation phases [31,32]. Improved anatomic resolution can be reached by integrating the electroanatomic maps with computer tomography scans or magnetic resonance imaging 3D reconstruction [33,34,35,36]. The CARTO-3 system is the last version of the system and allows the integration of magnetic fields with electrical fields such as those used by other EAM systems. EnSite NavX™ (St. Jude Medical, Abbott, Abbott Park, IL, USA) is a system based on impedance generated by electrical fields that, similar to CARTO, provides a point-to-point creation of the EAM [37]. A low-level current (1 mA) is applied through six patches positioned in orthogonal planes on the patient’s chest, and the potential difference is recorded by the catheter tip in order to determine its localization. The benefits of NavX are the compatibility of the system with all catheters and the acquirement point speed of 96 points/s, much faster than the CARTO system. Also, chamber geometry created by the NavX system can identify and automatically tag anatomical landmarks with a much higher resolution than that created by the CARTO system, in which points are manually acquired. This method provides EAMs based on several thousand points. EnSite X (Abbott) is a new mapping system combining impedance and magnetic field data to improve the real-time location of catheters. Integration with CT and MRI images is also possible. The RHYTHMIA Mapping System (Boston Scientific, Cambridge, MA, USA) is a hybrid system using both impedance and electrical fields combined with a multipolar basket-like catheter composed of high-density, very small-size electrodes. This allows the creation of ultra-high-resolution activation and voltage maps using rapid and accurate automated data acquisition and annotation [38]. Notably, RHYTHMIA does not allow for integration with CT or MRI. Table 1 provides an overview of the pros and cons of the above-cited EAM systems. EAM systems have simplified the diagnostic process and provided novel strategies for arrhythmias treatment [39]. A dramatic reduction in fluoroscopy time has been demonstrated without affecting the safety and efficacy of procedures [8,31]. On the other hand, intracardiac and thoracic impedance, as well as the patient’s movements during the procedure, can affect the map reconstruction and significantly prolong procedure times [40,41]. The employment of steerable catheters to improve ablation catheter handling and tissue contact, leading to reduced ablation time and high-quality lesions, is constantly increasing, particularly during pulmonary vein (PV) isolation [42]. The recent production of a new generation of steerable catheters that can be integrated with EAM systems and, therefore, visualized on the 3D map provided a significant optimization in the procedure workflow [43]. Their demonstrated use, compared to standard, non-visualizable catheters, further reduces the left atrial procedure time, RF delivery, and fluoroscopy exposure without compromising safety or effectiveness [44,45].

### 3.2. Intracardiac Echocardiography

ICE is performed by using a deflectable catheter carrying a two-dimensional ultrasound probe at the tip advanced through the femoral vein into the heart, providing high spatial and temporal resolution images of complex, moving structures such as valves and papillary muscles [46]. This imaging method precisely identifies tissue where ablation needs to be performed; obstacles like artificial prostheses and occluders are also visualized and avoided. ICE catheters can be divided into rotational catheters and phased-array catheters [10,47]. Radial ICE is composed of a tip with a single-rotating crystal element that provides a 360-degree view perpendicular to the longitudinal axis of the catheter and differs from phased-array ICE, which is made up of a 64-element transducer that allows for anteroposterior and lateral deflection. Phased-array catheters have wider applications because of their higher frequency range and ability of Doppler and color-flow imaging. The types of ICE systems available so far are Radial ICE (Ultra ICE™, Boston Scientific, Marlborough, MA, USA), ViewFlex™ Xtra ICE catheter (Abbott, Chicago, IL, USA), and ACUSON Acunav (J&J, New Brunswick, NJ, USA) [10]. ICE has been demonstrated to be a safe and feasible modality to provide localization of diagnostic and ablation catheters and real-time high-resolution images of cardiac structures with a significant reduction in fluoroscopy time exposure [48,49,50]. Unlike transesophageal echocardiography (TOE), ICE does not require sedation and can be performed by the same operator that performs the EP procedure [10]. Compared to fluoroscopy guidance, catheter placement is more difficult, especially for non-experienced operators. Nevertheless, a reduction in mapping and ablation time has been demonstrated [51,52]. ICE allows the detection of complex arrhythmogenic structures that are not visible with fluoroscopy, such as endocardial crypts, perivalvular tissues, ischemic scars, moderator bands, and papillary muscles. Moreover, ICE helps to exclude the presence of left atrial appendage sludge or thrombosis and provides direct visualization of the catheters, early detecting some potentially severe complications such as catheter thrombosis, pericardial effusion, and steam pop. ICE is considered a gold standard in the ablation of left-sided arrhythmias, in particular ventricular arrhythmias (VAs) [47,53,54]. Improved-quality images and fluoroscopy time reduction are obtained by integrating ICE with EAM, as made by the CARTOSOUND module by using a special phased-array ICE catheter (SOUNDSTAR ultrasound catheter, Biosense Webster, Irvine, CA, USA) [10,47]. Despite ICE having no potential contraindications and needing little vascular access (6–10 Fr), some factors, such as the need for experienced operators, the single-use label, and high costs, limit its spread. Caution is needed in patients with implanted cardiac devices because of the risk of lead dislocation [54]. Figure 1 shows the main benefits of fluoroless technologies.

## 4. Fluoroless Procedures in EP Labs

### 4.1. Paroxysmal Supraventricular Tachycardias

Supraventricular tachycardias (SVTs) are the most frequent cause of young individuals’ referral to the cardiologist or electrophysiologist because of discomfort and stress [55,56]. SVTs are registered from the gestation period to adulthood, and over 65 years old, the incidence increases five-fold. In the general population, the prevalence is 2.5 out of 1000 persons, and the incidence is 36 per 100,000 persons per year [57]; females are more prone to be affected [57], particularly during pregnancy [58]. Atrioventricular nodal re-entrant tachycardia (AVNRT), atrioventricular re-entrant tachycardia (AVRT), and atrial tachycardia (AT) are the most common paroxysmal SVTs. CA of this group of tachyarrhythmias is the gold-standard therapy due to its safety, high effectiveness, and cost savings [59]. The procedure is conventionally performed through fluoroscopy guidance, despite several meta-analyses reporting that they may be potentially performed completely fluoroless with similar results by using EAM systems [11,60]. Conflicting results exist in published studies about procedural times involving fluoroless ablations compared to fluoroscopy ones [26,27,61]. Overall, fluoroless was demonstrated to not prolong the procedure duration [62,63]. However, to achieve such results, a complete learning curve must be reached by the operators: Kochar et al. demonstrated that 20 procedures have to be performed to achieve the necessary confidence to reduce the fluoroscopic and the procedure time [64]. The acute procedure success of both methods is substantially similar [39,65]. The acute success rate was registered at above 97% [11], and only a few studies reported higher recurrence rates in the fluoroless group than in the conventional one [30]. No differences were reported in the complication rate [11]. Zero- or minimal-fluoroscopy CA plays an important role in the treatment of SVTs during pregnancy. In these cases, antiarrhythmic drugs may be effective but at costs of risk for the fetus [66], equal to radiation exposure. In these cases, fluoroless or minimal radiation exposure CA is considered mandatory [67,68], as reported in the latest guidelines with a recommendation level IIa and IIb, respectively [55,56]. Minimizing radiation exposure is also of paramount importance in the pediatric population to reduce long-term consequences [69,70].

### 4.2. Atrial Fibrillation and Atrial Flutter

AF and AFL are the most frequent cardiac arrhythmias, with a prevalence of 2% to 4%, and are showing continuous growth with higher prevalence in men and older people [71]. Accordingly, CA of these arrhythmias is the most performed EP procedure worldwide, being an effective strategy for rhythm control, leading to improved quality of life and mortality [72,73]. RF, electroporation, and cryoablation are the most commonly used methods. The mean radiation equivalent of an AF ablation performed with RF in conventional mode is 15 mSv [74]. The introduction of the EAM systems has been a turning point in the management of these arrhythmias as they improved the effectiveness of the procedure and the understanding of the persistent forms, as well as reduced the use of duodecapolar catheters. Moreover, since point-by-point AF ablation is a relatively longer procedure compared to single-shot techniques [10,75], achieving a reduction in fluoroscopic exposure was of paramount importance. Routinary use of echocardiography-guided transeptal puncture allows to reach the left atrium in a safer mode without fluoroscopy and is usually performed by TOE during deep sedation or general anesthesia. However, ICE may be an alternative echocardiographic guidance in those patients who need lesser deep sedation without the risk of esophageal complications [76].

As reported in a recent meta-analysis, fluoroless AF ablation reduces the fluoroscopy time (−5.21 min; −5.51, −4.91; *p*-value < 0.01) and the radiation dose (−3.96 mGy; −4.27, −3.64; *p*-value < 0.01) in a consistent manner without influencing ablation time [5]. Accordingly, a recent retrospective study reported a procedure time of 176 ± 46 vs. 194 ± 56 min compared to conventional AF ablation (*p*-value = 0.0021) [77]. Among different EAM systems, those based on magnetic fields have been linked to shorter time with significant reductions in fluoroscopy and RF delivery due to their relative independence from impedance changes linked to tissue edema, respiration, and periprocedural fluid shift [78]. Single-shot technologies were developed to standardize the ablation workflow and reduce times but do not use mapping systems and, therefore, remain relatively dependent on fluoroscopy so far. A first attempt to perform fluoroless PV cryoablation by using ICE was performed in 2021 by Alyesh et al. [79] and recently reproduced in a randomized study by Janhee et al. [80]. The indicators of PV occlusion used in the studies included the integration of hemodynamic measures with continuous-wave pressure monitoring and ICE color Doppler images in the first and ICE alone in the second. Compared to the conventional approach, the fluoroless group did not differ in procedural time, acute success, complication, and recurrence rate. Compared to AF, CA of AFL is very effective in definitively abolishing the arrhythmia, with a rate of recurrence <10% for the most common type of AFL, which is dependent on a macro re-entry around the cavotricuspid isthmus (CTI) [81]. Atypical AFLs are the minor part and include those arising in the left atrium and are usually associated with cardiomyopathies, cardiac surgery, and incomplete AF ablation. CA of these types are longer and more complex, with a significantly higher rate of recurrence [82]. AFL ablation guided by EAM systems aims to identify the best line of ablation based on local electrograms and activation map of the re-entry circuit, then to assess the bidirectional block. Compared to conventional ablation, this method demonstrated similar efficacy and procedure time but avoided X-ray exposure at the expense of increased costs [83,84]. AFL ablation guided by ICE alone was also explored in some studies, reporting an improved success rate, decreased procedure and ablation time, and minimized radiation exposure due to a better visualization of the CTI [85,86,87]. Finally, the integration of ICE and 3D EAM allows the performance of a safe and precise transeptal puncture as well as provides high-resolution imaging of the anatomy of both right- and left-sided cardiac structures, including LAA. With this method, all types of AFL are potentially approachable without the use of fluoroscopy and without compromising duration, safety, or efficacy [53,88].

### 4.3. Ventricular Tachycardia and Premature Ventricular Contraction

VAs range from isolated or clustered PVC to potentially lethal sustained VT and ventricular fibrillation (VF). The incidence of lethal VAs is approximately 50 per 100,000 person-years in middle-aged individuals and increases with age [89]. CA is demonstrated to be effective in the treatment of the major part of VAs and is generally recommended in cases of recurrent VAs leading to left ventricular dysfunction, failure of antiarrhythmic drugs, or multiple interventions of implantable cardioverted defibrillator [89]. Only case reports and observational studies have been published so far regarding zero-fluoroscopy CA of VAs [90,91,92]. EAM systems are deemed necessary with these types of arrhythmias due to the higher accuracy in the identification of the ablation substrate, the additional insights provided on VA electrical behavior, and the need in some cases to perform ablation in structures in continuous movement. The combined use of EAM systems and ICE also provides advantages over the conventional procedure due to the capacity to add an anatomical view of the target structure that may be of paramount importance in cases of high-movement structures such as papillary muscles or high-risk structures such as the left ventricular summit due to its proximity to the coronary vessels [90]. ICE also provides the real-time thickness of the wall, which is important in deciding the amount of energy to use; this has particular importance for the ventricle wall, which varies from 3 mm to 25 mm [93]. Possible complications can also be recognized early [91]. Important technical concerns of VA ablation are related to the frequent need for multiple accesses to cardiac chambers (either transeptal or retroaortic) that significantly prolong the procedure time. Furthermore, patients with complex, severe VAs are often affected by advanced heart failure and multiple comorbidities; therefore, they cannot tolerate excessively prolonged procedures. On the contrary, those with idiopathic VAs are usually younger, fit, and with normal hearts; fluoroless procedures may fit very well in this population. Lamberti et al. [91] enrolled nineteen patients who underwent zero-fluoroscopy idiopathic VA ablation, including VT and PVCs from the right ventricle outflow tract (RVOT, 42%), the left ventricle outflow tract (LVOT, 21%), the left fascicle (16%), the peri-tricuspidalic region (11%), the peri-mitral region (5%) and the lateral left free wall (5%). The acute success rate was 100% without complications reported, suggesting that the fluoroless ablation is possible in a wide variety of regions, with both a retrograde transaortic or a transeptal puncture approach. Similar results were collected in a previous study on a pediatric population [94]. More recently, Sadek et al. [92] reported successful zero-fluoroscopy VA ablation in four subjects with idiopathic VAs and six subjects with structural heart disease. ICE imaging was substantial for guiding the catheters in device carriers. Accordingly, a learning curve of 15–20 cases is deemed necessary to carry out the procedure in normal times [64]. Major studies on fluoroless ablation are described in Table 2.

## 5. Device Implantation and Other Possible Use of Zero-Fluoroscopy

X-ray guidance is currently the gold standard for every cardiac pacing procedure, despite few studies and case reports reporting some alternative options to be considered in special cases like pregnancy and childhood. The CARTO system was used to place an atrial catheter in a patient with Ebstein’s anomaly for the corrected placement of the catheter in a dilated right atrium with a diffused low-amplitude voltage signal [95]. EnSite NavX was used to guide the implantation of a single-lead atrioventricular pacemaker in fifteen patients [96]. Similar cases have been described in the context of AF treated with ablate and pace [97] and in a case series of CRT-D implants [98]. To cannulate and map the coronary sinus (CS), an electrophysiological catheter connected to an EAM system was introduced via the subclavian vein and was used to create the 3D map of the CS branches. When necessary, especially for the smallest vessels, wire cannulation was performed using fluoroscopy. During vessel mapping, the local ventricular activation time and the bipolar voltage amplitude were recorded using the right ventricular electrogram as a reference. The final position of the CS lead was chosen by relating the maximum activation delay between the electrogram in the right ventricle and the electrogram in the CS branch. Transthoracic ultrasound guidance and modification in radiation protocols, like ultralow frame rate at 2–4 frame/s, could otherwise reduce the radiation exposure without a significant increase in procedure time [99,100]. Recently, EAM systems application in guidance of percutaneous endomyocardial biopsy (EMB) to identify myocardial pathological substrate demonstrated to be feasible and to improve precision and diagnostic yield of the biopsy [101]. Of note, a cutoff of 5 mV voltage amplitude demonstrated a substantially higher sensitivity (70% vs. 26%) and a negative predictive value (62%) than 1.5 mV in predicting abnormal myocardium. Compared to CMR, electrogram-guided EMB showed similar sensitivity and good specificity in detecting myocardial scar areas [102].

## 6. Cost-Effectiveness

The use of 3D mapping systems and ICE involves an important amount of costs compared to fluoroscopy [103]. Some studies have been conducted to evaluate if these technologies are cost-effective in terms of long-term reduction in radiation complications. The cost-effectiveness of these procedures has been demonstrated only in some cases, such as children [104], and in cases of complex CA necessitating very high fluoroscopy use, such as for AF ablation [105]. Traditional SVT ablation requires a lesser amount of radiation; therefore, fluoroless procedures are currently considered only in cases where X-ray use is avoided. A more extensive analysis must be conducted, taking into account the financial advantage that zero-fluoroscopy provides not only in reducing patients’ exposure to radiation but operators’ too, including prevention of orthopedic complications. In the future, with the aim to reduce waste, costs, and environmental impact, the reprocessing of single-use catheters and other electrophysiological tools will have to be considered. A study reported the feasibility and safety of ICE probe reprocessing that allowed the use of the same probe up to 20 times, resulting in 90% cost reduction (>EUR 2 million in savings for the studied period) and 95% waste reduction (639.5 kg less, mostly non-degradable) without increased risk of infection and malfunction [106]. Nevertheless, currently available single-use devices are not approved for multiple use, and universally accepted guidelines that regulate device sterilization and reprocessing are lacking. In Brazil, the reprocessing of such products is regulated by the National Health Surveillance Agency (ANVISA), which demands manufacturers that label their products as single-use to submit documents that substantiate the reasons for not reprocessing. Currently, the list provided by ANVISA on medical products whose reprocessing is invariably forbidden does not contain any product used in the electrophysiological procedures routine [107]. This could be a starting point to extend the concept of cost and waste saving to a universal level.

## 7. Limitations and Future Perspectives

The topic of radiation exposure is of paramount importance because of the increase in life expectancy and the use of X-rays in medical practice. In the EP field, the “Go for Zero Fluoroscopy” project is working to find valuable alternatives to X-rays. A 2020 registry review enrolling 25 EP laboratories from 14 European countries reported a hopeful trend toward a reduction in radiation, carried out in particular by higher volume centers [108]. However, the importance of a readily available C-arm cone beam in EP laboratories has to be highlighted for its rapid accessibility, easy interpretation of fluoroscopic images, and long experience. Its readiness is of paramount importance in cases of procedural complications such as cardiac tamponade, vascular complications, anatomical variants, and technical problems of the EAM system. This condition also raises the question of when X-ray aprons can be avoided. So far, it is advisable to wear X-ray aprons in cases of complex CA involving procedures at higher risk of severe complications like transeptal or epicardial puncture and the retroaortic approach. Moreover, fluoroscopic support can be helpful since EAM systems are not able to identify intracardiac obstacles such as electrocatheters already in place. Fluoroless procedures may be improved in the next future by advancements in several technological fields: (I) imaging technologies such as advanced ultrasound, CMR, and 3D EAM systems integrated with artificial intelligence algorithms for better procedural guidance; (II) increased employment of robotics and automation in EP procedures could improve precision and reduce the reliance on fluoroscopy; and (III) advancements in personalized treatment plans involving pre-procedural imaging, simulation tools, and advanced machine learning to improve the effectiveness and safety of more complex procedures. Finally, the operator’s experience and mindset are a fundamental factor to be considered in order to reduce X-ray exposure. Indeed, high use of fluoroscopy is always reported at the beginning of the training, with its reduction achieved with increasing experience [109]. Extensive training is therefore needed to improve operators’ skills; simulators can be used to practice and become accustomed to the procedure. A solid experience in conventional procedures is also needed in order to use fluoroscopy as low as possible if needed. Of paramount interest, it has been demonstrated that being a female operator is an independent predictor for low radiation exposure [110].

## 8. Conclusions

Radiation exposure in EP lab is an utmost important topic and deserves further consideration and diffusion in the future. The protection of both patients and operators has to be taken into serious account due to the negative effects that long-term X-ray exposure can cause. Novel echocardiographic and electroanatomic imaging modalities paved the way for fluoroless procedures in high-volume centers. However, high costs and the need for experienced operators currently limit their diffusion in peripheral ones.

## Figures and Tables

**Table 1:** Pros and cons of above-cited EAM systems.

	CARTO	Ensite NavX	RHYTHMIA
Company	Biosense Webster	Abbott	Boston Scientific
Location method	Magnetic field	Electrical and magnetic field	Electrical field
Mapping technique	Point-to-point	Point-to-point	Non-contact
Compatibility with any catheter	No	Yes	No
Patient movement sensibility	High	Low	Low
Need for intracardiac reference catheter	No	Yes	Yes
Contact-sensing catheters	Force	Electromagnetic	Impedance
CT–CMR integration	Yes	Yes	No
ICE integration	Yes	No	No
Pros	- Anatomical accuracy - Imaging integration - Contact force sensing catheters	- Anatomical accuracy - Different mapping catheters allowed - Imaging integration - Less arrhythmogenic shape of multipolar mapping catheter	- Mapping beat to beat
Cons	- Biosense Webster catheters - Sensibility to patient movements	- Sensibility to reference catheter movement - Absence of contact sensibility on multipolar mapping catheters	- Less precise anatomical map - Stiffer catheters - Absence of contact sensibility on multipolar mapping catheters

Abbreviations: CMR, cardiac magnetic resonance; CT, cardiac tomography; ICE, intracardiac echocardiography.

**Table 2:** Major studies in fluoroless ablation.

Reference	Arrhythmia Treated	No. of Patients (Fluoroless vs. Conventional Fluoroscopy)	Main Findings
Di Cori et al. [61]	SVT/AFL	93 vs. 116	- Safety and efficacy of fluoroless - Arrhythmia type predicted fluoroless procedure
Kalinsek et al. [16]	SVT	294 vs. 280	- Safety and efficacy of fluoroless in adult and pediatric populations
Bergonti et al. [30]	SVT	206 vs. 412	- Better long-term results and reduced complications with fluoroless
Casella et al. [39]	SVT	134 vs. 128	- Safety and efficacy of fluoroless - Reduction in patients’ exposure, risk of cancer and mortality
Stec et al. [62]	SVT	179 vs. 714	- Safety and efficacy of fluoroless - No difference in procedure time, complication rate, acute and long-term success
Chen et al. [63]	SVT	1020 vs. 2040	- Safety and efficacy of fluoroless - Reduction in radiation exposure
Fadhle et al. [65]	SVT	100 (Carto) vs. 100 (Ensite) vs. 100	- Safety and efficacy of EAM systems
Ferguson et al. [76]	AF	21	- Feasibility of fluoroless, in particular in childhood, pregnancy, and obesity
Lurie et al. [77]	AF	147 vs. 176	- Safety and efficacy of fluoroless - Reduced procedure times - Similar acute success, complication rate, and recurrence
Khaykin et al. [78]	AF	71 (Carto) vs. 165 (Ensite) vs. 197	- Lower procedure time, fluoroscopy duration, and radiofrequency energy delivery time with EAM systems
Rivera et al. [90]	TV/PVC	27	- Safety and efficacy of fluoroless - Acute success rate 84%, recurrence rate 24%
Lamberti et al. [91]	TV/PVC	52	- Safety and efficacy of fluoroless - 100% acute success rate
Sadek et al. [92]	AF/AFL/VT	80	- Safety and efficacy of fluoroless - No increase in procedural time - Medium learning curve
Alyesh et al. [79]	AF	50 vs. 50	- Safety and efficacy of fluoroless cryoablation
Jinhee et al. [80]	AF	50 vs. 50	- Safety and efficacy of fluoroless, ICE-guided procedure
Turcsan et al. [86]	AFL	219 vs. 151	- Safety and efficacy of fluoroless, ICE-guided procedure - 100% acute success rate - Shorter procedure time
Jacinto et al. [88]	AFL	31 vs. 191	- Safety and efficacy of fluoroless - Reduction in procedure time

Abbreviations: AF, atrial fibrillation; AFL, atrial flutter; EAM, electroanatomic mapping system; SVT, supraventricular tachycardia; PVC, premature ventricular contraction; VT, ventricular tachycardia.

## Data Availability

Not applicable.
